# An Entry-Triggering Protein of *Ehrlichia* Is a New Vaccine Candidate against Tick-Borne Human Monocytic Ehrlichiosis

**DOI:** 10.1128/mBio.00895-20

**Published:** 2020-07-28

**Authors:** Khemraj Budachetri, Omid Teymournejad, Mingqun Lin, Qi Yan, Mariella Mestres-Villanueva, Guy Nathaniel Brock, Yasuko Rikihisa

**Affiliations:** a Department of Veterinary Biosciences, College of Veterinary Medicine, The Ohio State University, Columbus, Ohio, USA; b Center for Biostatistics, The Ohio State University, Columbus, Ohio, USA; Brigham and Women's Hospital

**Keywords:** *Ehrlichia chaffeensis*, EtpE, ISCOM, interferon gamma, dogs, human monocytic ehrlichiosis, immunization, neutralizing antibodies, tick cells, tick-borne pathogens

## Abstract

Ehrlichia chaffeensis is an obligatory intracellular bacterium that causes human monocytic ehrlichiosis, an emerging disease transmitted by the Lone Star tick, Amblyomma americanum*. E. chaffeensis* outer membrane protein entry triggering protein of *Ehrlichia* (EtpE) is necessary for bacterial entry into human cells. We investigated the role of EtpE in transmission of the bacteria from tick to human cells and whether or not vaccination with EtpE can prevent transmission of ehrlichiae from ticks to mammals. An antiserum against the recombinant C terminus of EtpE (rEtpE-C), which binds a mammalian cell-surface receptor and triggers bacterial entry, significantly inhibited *E. chaffeensis* transmission from infected tick cells to human monocytes in culture. Each of five specific-pathogen-free dogs were vaccinated with rEtpE-C along with an immunostimulating complex or were sham vaccinated with the complex alone. Dogs vaccinated with rEtpE-C developed high antibody titers against rEtpE-C and produced interferon-γ-secreting cells, as assessed with the ELISpot assay. All 10 dogs were challenged with *A. americanum* adult ticks infected as nymphs by syringe inoculation with *E. chaffeensis*. Upon challenge, both the vaccinated and control dogs became infected by day 1 post-tick attachment, but the majority of rEtpE-C-vaccinated dogs rapidly cleared the infection from the bloodstream as soon as day 7, whereas most of sham-vaccinated dogs remained infected at day 35. Peripheral blood leukocytes from vaccinated dogs had significantly elevated interferon-γ mRNA levels and secreted significantly elevated interferon-γ soon after tick attachment. Thus, the EtpE-C vaccine represents the first ehrlichial protein vaccine demonstrated to reduce bacterial infection in mammals upon challenge with infected ticks.

## Abstract (precis)

The incidence of tick-borne diseases has risen dramatically in the past two decades and continues to rise. Discovered in 1986 and designated a nationally notifiable disease in 1998 by the Centers for Disease Control and Prevention, human monocytic ehrlichiosis, which is caused by the bacterium Ehrlichia chaffeensis, is one of the most prevalent, life-threatening, emerging tick-borne zoonoses in the United States. We investigated the role of the *E. chaffeensis* protein EtpE in transmission of the bacterium from tick to human cells and in vaccinated dogs with EtpE to assess the efficacy of vaccination against *E. chaffeensis*-infected tick challenge. Our results help fill gaps in our understanding of *E. chaffeensis*-derived protective antigens that could be used in a candidate vaccine for immunization of humans to counter tick-transmitted ehrlichiosis.

## INTRODUCTION

Ehrlichia chaffeensis is an obligatory intracellular bacterium that replicates within human blood monocytes and causes the emerging tick-borne infectious disease human monocytic ehrlichiosis (HME), which is characterized by severe systemic flu-like illness with hematologic abnormalities and mild hepatitis. HME can have relatively severe effects on older adults and persons with underlying health conditions and/or immunocompromised individuals. HME is often undiagnosed or misdiagnosed owing to nonspecific clinical signs and/or the lack of specific, sensitive, and readily available diagnostic tests, particularly at early stages of infection. The current therapy of choice is the broad-spectrum antibiotic doxycycline, which is effective only if initiated early because any delay in initiating therapy can lead to severe sepsis-like complications or death with a mortality rate of 2% to 5% (1). No vaccines exist for HME.

The Lone Star tick (Amblyomma americanum) serves as the primary biological vector for *E. chaffeensis* (2), and *E. chaffeensis* DNA has been detected in *Amblyomma* sp. and related tick species in regions of HME endemicity worldwide (3–5). The Lone Star tick is an aggressive nonspecific feeder and bites humans at all three developmental stages, i.e., larvae, nymph, and adult. In fact, when 222 *A. americanum* ticks removed from humans were tested, 33 (15%) had *E. chaffeensis* DNA, indicating a high chance of transmission from infected ticks to humans (6). White-tailed deer (Odocoileus virginianus) are well-known natural blood reservoirs for *E. chaffeensis* (7, 8), in addition to serving as important hosts to all three mobile stages of the Lone Star tick (9). These deer have been overpopulated for decades in much of the continental United States, contributing to the emergence and expansion of HME (10).

*E. chaffeensis* has a small genome (1.2 Mb) and lacks primary pathogen-associated molecular patterns, such as lipopolysaccharide (an endotoxin), peptidoglycan, flagella, pili, and a capsule, as well as exotoxins (11, 12). The essential step in *E. chaffeensis* virulence is its entry into eukaryotic host cells, wherein it replicates by hijacking/dysregulating cell functions. The survival of *E. chaffeensis* is secured only by its specific mode of entry, which is mechanistically distinct from phagocytosis (13). Our recent studies showed that the unique *E. chaffeensis* surface-exposed outer membrane protein entry triggering protein of *Ehrlichia* (EtpE; ECH1038, GenBank accession number YP_507823 for Arkansas^T^) functions as an invasin (13). EtpE is highly expressed during the intracellular *E. chaffeensis* developmental stage called the dense-cored cell, which precedes *E. chaffeensis* release from host cells to initiate a new cycle of infection (14). The C-terminal region of EtpE (EtpE-C) is absolutely conserved among *E. chaffeensis* strains, and this region extends outwardly from the bacterial surface. We previously produced a recombinant EtpE-C (rEtpE-C; 308 residues) and used EtpE-C-coated latex beads to demonstrate that this C-terminal portion alone could mediate the invasion of host cells, whereas the N-terminal portion (anchored in the *E. chaffeensis* outer membrane) could not (13, 15). We discovered that the mammalian cell-surface glycosylphosphatidyl inositol-anchored protein DNase X (DNase-1-like 1) is the receptor for EtpE-C-mediated entry. DNase X directly binds EtpE-C, antibody-mediated neutralization of DNase X or small interfering RNA (siRNA)-mediated suppression of its expression could impair the binding and entry of *E. chaffeensis* and rEtpE-C-coated beads, and consequently host-cell infection was prohibited (13). Furthermore, DNase X knockout (DNase X^–/–^) in mice significantly reduced the bacterial load in both whole animals and macrophages derived from them (13), pointing to a key role for EtpE-C-mediated entry via DNase X in *E. chaffeensis* infection.

EtpE is expressed by *E. chaffeensis* in HME patients (naturally infected by a tick bite) and in dogs infected experimentally, as evidenced by the production of specific antibodies against EtpE (13). EtpE is essential for the infection of monocytes because an antibody against rEtpE-C could greatly inhibit *E. chaffeensis* binding, entry, and infection. Moreover, vaccination of mice with rEtpE-C significantly inhibits *E. chaffeensis* infection upon intraperitoneal *E. chaffeensis* challenge (13), suggesting that humans at risk for HME could also be similarly vaccinated. Therefore, we examined whether a polyclonal anti-rEtpE-C serum could block the transmission of *E. chaffeensis* from tick cells to human monocytes in culture. Moreover, the dog is naturally infected with *E. chaffeensis* (16–18) and can serve as a useful animal model for tick transmission of *E. chaffeensis* (19, 20). Thus, we examined whether or not vaccination of dogs with rEtpE-C could reduce the incidence of tick-mediated transmission of *E. chaffeensis* to dogs.

## RESULTS

### An antibody against rEtpE-C inhibits the transmission of *E. chaffeensis* from tick cells to human monocytes.

**FIG 1:**
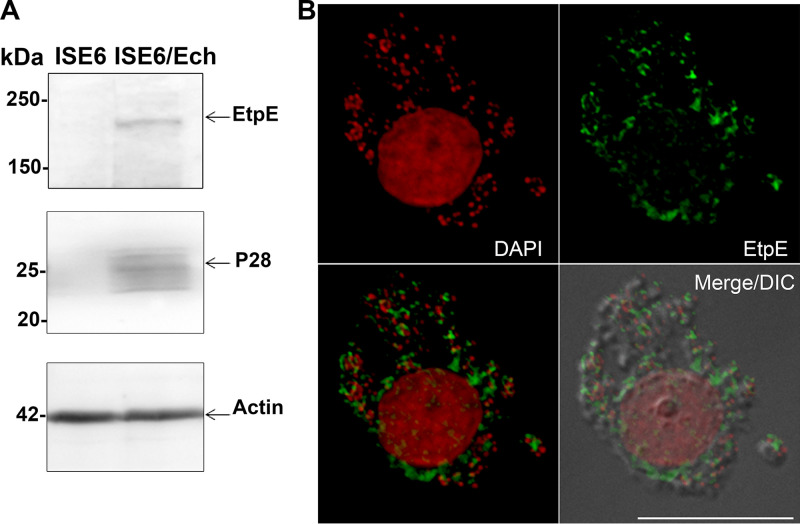
EtpE is expressed by *E. chaffeensis* in tick cells. (A) Expression of native EtpE by *E. chaffeensis* in ISE6 cells. *E. chaffeensis-*infected ISE6 cells at 3 days postinfection were subjected to Western blotting with rabbit anti-rEtpE-C. Infection of cells with *E. chaffeensis* was assessed with anti-P28 and normalized by I. scapularis tick actin. (B) *E. chaffeensis-*infected ISE6 cells at 3 days postinfection were labeled with anti-rEtpE-C and Alexa Fluor 488-conjugated anti-rabbit IgG. *E. chaffeensis*, and host DNAs were labeled with DAPI (pseudocolored red). Merge/DIC, fluorescence images merged with differential interference contrast images with a DeltaVision microscope. Scale bar, 10 μm.

A global transcriptome analysis (21) revealed that the pattern of gene expression in *E. chaffeensis* differs depending on whether or not the bacterium infects tick cells or mammalian cells. *E. chaffeensis* grown in tick cells expresses proteins that best fit the tick environment. For effective tick-to-human transmission, however, *E. chaffeensis* must be liberated from tick cells and rapidly enter human cells because the bacterium cannot survive outside a eukaryotic cell. Once it is inside a mammalian cell, *E. chaffeensis* gene expression is reprogrammed. We previously showed that EtpE is expressed by *E. chaffeensis* cultured in several mammalian cells, including human primary macrophages derived from peripheral blood monocytes and that native EtpE can be identified in Western blots and observed via immunofluorescence microscopy using an antiserum against rEtpE-C (13). In the tick cell lines AAE2 and ISE6 infected with *E. chaffeensis*, *EtpE* mRNA is expressed at higher levels than in the *E. chaffeensis*-infected human monocytic leukemia cell line THP-1, as assessed with microarrays (21). This suggests that EtpE is critical for transmission from a tick to mammalian cells at the site of a tick bite, and thus, it is possible that anti-rEtpE-C could be used to inhibit tick-to-human transmission of *E. chaffeensis* by inhibiting entry of tick cell-resident *E. chaffeensis* into mammalian cells. To test this possibility, we first confirmed that EtpE is indeed expressed by *E. chaffeensis* in cultured ISE6 cells, as assessed with Western blotting and immunofluorescence microscopy (Fig. 1).

**FIG 2:**
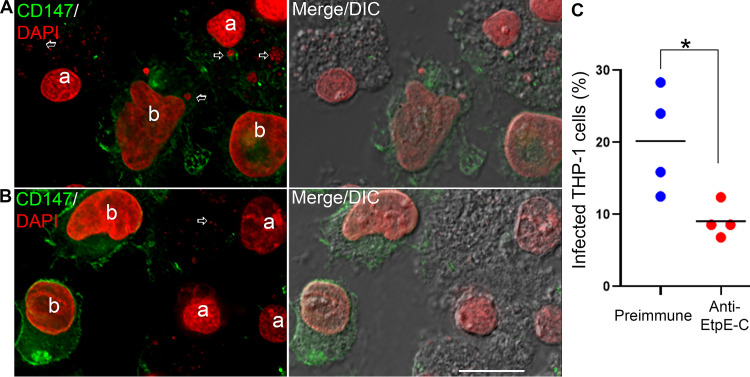
Anti-EtpE-C inhibits the transmission of *E. chaffeensis* from tick cells to human monocytes. Infected ISE6 cells (a) were incubated with uninfected THP-1 cells (b) in the presence of rabbit preimmune (A) or anti-EtpE-C (B) serum for 2 days. The cocultured cells were labeled with mouse anti-human CD147 (green). Bacteria and the host cell DNA were labeled with DAPI (pseudocolored red). (C) Quantitation of 200 THP-1 cells each in four independent experiments. *, *P < *0.05, based on the unpaired two-tailed Student’s *t* test. Horizontal bars indicate mean values.

A transmission blocking assay was designed to assess the ability of anti-rEtpE-C serum to prevent *E. chaffeensis* transmission from infected ISE6 cells to uninfected human THP-1 monocytes. *E. chaffeensis-*infected tick cells were cocultured with uninfected THP-1 cells in the presence of anti-rEtpE-C serum or preimmune serum for 2 days, and the percentage of infected THP-1 cells was scored. Although ISE6 and THP-1 cells could be easily differentiated based on cell size and nuclear shape (Fig. 2A and B), to unambiguously distinguish these two cell types, we used an antibody against human CD147, which specifically labels THP-1 cells, and 4′,6-diamidino-2-phenylindole (DAPI), which labels the nucleus of both host cells and *E. chaffeensis*. The immunofluorescence microscopy results revealed that anti-rEtpE-C significantly inhibited the transmission of *E. chaffeensis* from infected ISE6 cells to cocultured THP-1 cells (Fig. 2C).

### EtpE is expressed by *E. chaffeensis* in adult *A. americanum* ticks infected as nymphs.

**FIG 3:**
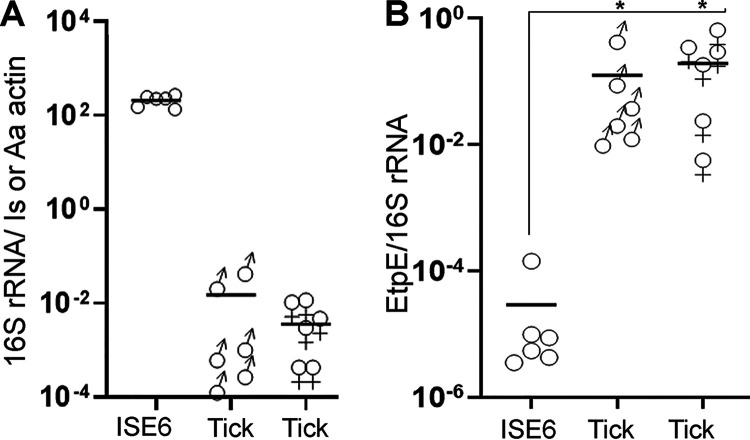
EtpE is expressed by *E. chaffeensis* in adult *A. americanum* ticks infected as nymphs. (A) Expression of 16S rRNA by *E. chaffeensis* in ISE6 cells or in molted male and female *A. americanum* ticks normalized by I. scapularis (Is) actin (ISE6) mRNA or *A. americanum* (Aa) tick actin mRNA, respectively. (B) Expression of *EtpE* mRNA by *E. chaffeensis* in ISE6 cells and in *A. americanum* ticks normalized by *E. chaffeensis* 16S rRNA (RT-qPCR). *, *P < *0.0001, based on ANOVA. Horizontal bars indicate mean values.

The tick *A. americanum* is the primary biological vector for *E. chaffeensis* replication and transmission (10). *A. americanum* are three-host ticks, taking the first and second blood meals from different hosts during the larval and nymphal stages to molt into the next stage (22). To prepare *E. chaffeensis*-infected ticks for our experimental transmission study, 1,150 freshly engorged *A. americanum* nymphs were needle injected with *E. chaffeensis* (7× 10^8^ to 10 × 10^8^ bacteria in 2 to 4 μl per tick) freshly isolated from infected DH82 cells. The engorged, injected nymphal ticks were allowed to molt in an incubator, resulting in 400 males and 680 female adult ticks (molting efficiency, 94%). *E. chaffeensis* infection in the molted adult ticks was verified by reverse transcription-quantitative PCR (RT-qPCR) of *E. chaffeensis* 16S rRNA. All tested ticks (*n* = 40; 25 females, 15 males) were infected with *E. chaffeensis*, indicating effective transstadial transmission (Fig. 3A). The number of bacteria in whole ticks was much lower than that in ISE6 cell cultures when normalized with *A. americanum* tick actin and Ixodes scapularis tick actin (ISE6 cells), respectively, most likely due to variations in the amounts of bacteria in various tick tissues (Fig. 3A). However, expression of *EtpE* mRNA by *E. chaffeensis*, which was normalized to that of *E. chaffeensis* 16S rRNA, was significantly greater in both female and male ticks than in ISE6 cells, as assessed with RT-qPCR (Fig. 3B).

### Dogs vaccinated with rEtpE-C develop high antibody titers against rEtpE-C and clear *E. chaffeensis* rapidly upon challenge with *E. chaffeensis*-infected ticks.

Specific-pathogen-free Beagle dogs (age, 1 to 2 years; 10 dogs, 4 male, 6 female) were vaccinated with rEtpE-C and the immunostimulating complex (ISCOM) (2 male and 3 female) or with ISCOM alone (sham vaccination control; 2 male and 3 female) three times at 2-week intervals as described in the Materials and Methods. The ISCOM is a spherical open cage-like structure (30 to 40 nm in diameter) that forms spontaneously when antigens are mixed together with cholesterol, phospholipids, and Quillaia saponins (23). The complex stimulates the immune system and is often included in a vaccine to induce a stronger immune response and hence longer-lasting protection (24).

At 14 to 24 days after the last vaccination, two tick-feeding chambers made of stockinettes were attached to the skin of each dog, and 30 *E. chaffeensis-*infected ticks (20 females, 10 males) were placed in each stockinette. Mating occurs on the host upon the third blood meal, and both male and female ticks were included in each stockinette because mating promotes feeding on blood (25). The male must feed to produce spermatophores, and the female must feed to produce eggs (22). Ticks were allowed to feed for 13 to 15 days until ticks started to drop, and the remaining ticks were pulled off manually. These transmission-fed ticks were dissected and analyzed for *E. chaffeensis* load and EtpE expression in their salivary glands. Clinical signs and rectal temperature of dogs were monitored daily, and blood parameters (complete blood count with white blood cell differential and serum chemistry) were analyzed before the first vaccination, after the third vaccination, and at 1 and 3 weeks after tick challenge. Antibody development in the dogs was determined in sequentially collected blood samples by enzyme-linked immunosorbent assay (ELISA) and Western blotting. RNA was extracted from the blood samples, and *E. chaffeensis* abundance was determined by RT-qPCR of the *E. chaffeensis* 16S rRNA gene. The production of interferon-γ (IFN-γ) and other cytokines in blood samples was also measured by RT-qPCR.

**FIG 4:**
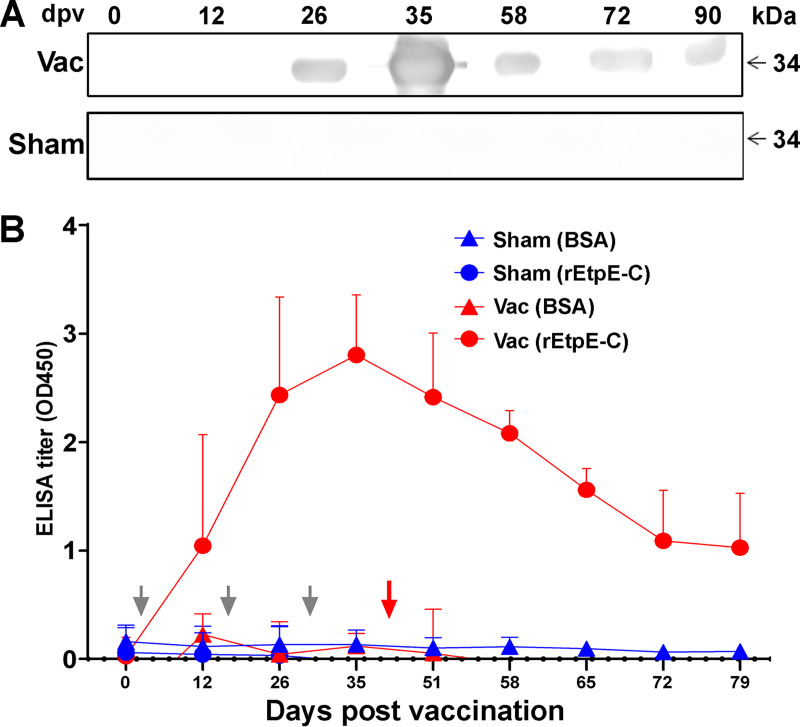
Dogs vaccinated with rEtpE-C develop high antibody titers against rEtpE-C. (A) Western blotting for rEtpE-C of plasma from a vaccinated dog (top) and a sham-vaccinated dog (bottom). dpv, days postvaccination. Shown are representative results from five vaccinated and five sham-vaccinated dogs. (B) ELISA titers (optical density at 450 nm [OD_450_]) using rEtpE-C or BSA (negative control) as the antigen. Vac, rEtpE-C-vaccinated. Sham, sham-vaccinated. Shown are results (mean ± SD) from five vaccinated and five sham-vaccinated dogs. Gray arrows indicate the days on which dogs were vaccinated, and the red arrow denotes the day on which the infected ticks started to attach.

**FIG 5:**
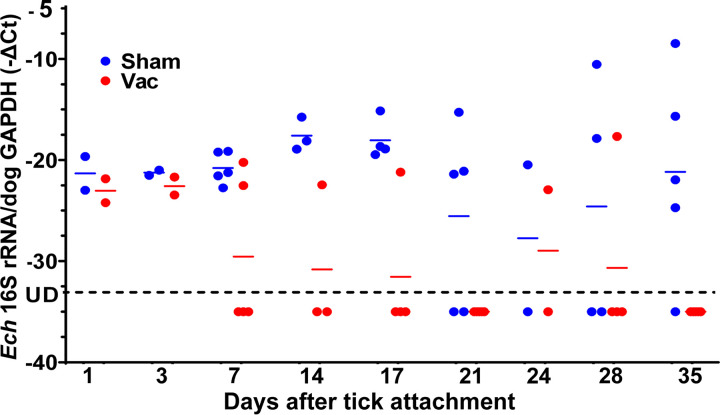
Dogs vaccinated with rEtpE-C rapidly clear *E. chaffeensis* infection. –Δ*C_T_* (cycle threshold) is –[*E. chaffeensis* load (*C_T_* value of *E. chaffeensis* 16S rRNA – *C_T_* value of dog *GAPDH* mRNA)] in peripheral blood from rEtpE-C-vaccinated and sham-vaccinated dogs after attachment of *E. chaffeensis-*infected ticks (RT-qPCR). *Ech*, *E. chaffeensis*; UD, undetectable (RT-qPCR). The difference between the rEtpE-C-vaccinated and sham-vaccinated dogs across all days postinfection was significantly different (*P = *0.0017), as assessed with the censored-data Cox model with random effects.

No significant clinical signs, fever, or abnormalities in blood cell counts or chemistry were detected throughout the study. Substantial antibody titers specific to rEtpE-C were attained for all vaccinated dogs but not for any of the sham-vaccinated dogs, as assessed with Western blotting (Fig. 4A) and ELISA (Fig. 4B) throughout the study. The *E. chaffeensis* burden in the blood was monitored until 35-days post-tick attachment (Fig. 5). Differences in bacterial 16S rRNA (normalized by dog blood cell *GAPDH* mRNA) between sham-vaccinated dogs (5 dogs) and rEtpE-C-vaccinated dogs (5 dogs) were statistically analyzed for samples taken at various days post-tick challenge. Cycle threshold (*C_T_*) values of >45 were capped at 45 and treated as censored values. Survival analysis using a censored-data Cox model (the “coxme” function) was performed to account for capped (censored) values with random effects for dogs to account for repeated measures (26). The difference between vaccinated and sham-vaccinated dogs across all days postinfection was statistically significant (*P = *0.0017), indicating a significant reduction in bacteria in the blood of vaccinated dogs compared with sham-vaccinated dogs (Fig. 5).

### IFN-γ is induced in rEtpE-C-vaccinated dogs.

**FIG 6:**
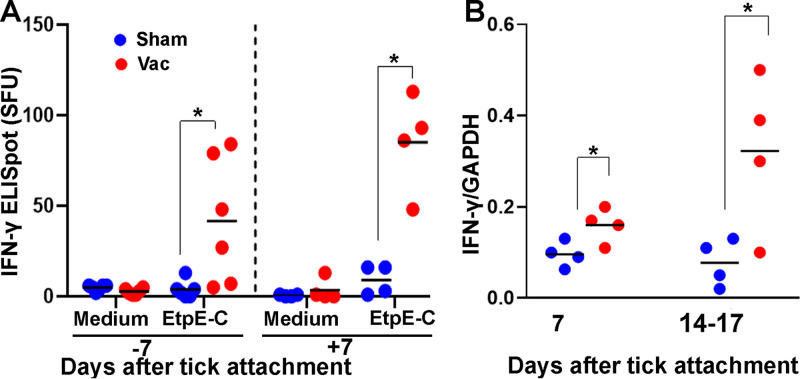
Vaccination of dogs with rEtpE-C induces a rapid IFN-γ response to *E. chaffeensis* challenge. (A) Canine IFN-γ ELISpot assay. PBMCs were isolated from two rEtpE-C-vaccinated and two sham-vaccinated dogs 7 days before and 7 days after the *E. chaffeensis-*infected ticks became attached. PBMCs were incubated with EtpE-C or medium (negative control) in triplicates. ELISpot spot-forming units (SFUs) were quantified with an ImmunoSpot analyzer, and the data from two dogs in each group were analyzed for differences between rEtpE-C-vaccinated and sham-vaccinated dogs using a negative binomial mixed model (*, *P < *0.001). (B) Expression of IFN-γ mRNA normalized by dog GAPDH mRNA in blood samples from four rEtpE-C-vaccinated and four sham-vaccinated dogs on day 7 and days 14 to 17 after the attachment of *E. chaffeensis-*infected ticks (RT-qPCR). *, *P < *0.05; based on the unpaired two-tailed Student’s *t* test. Horizontal bars indicate mean values.

**FIG 7:**
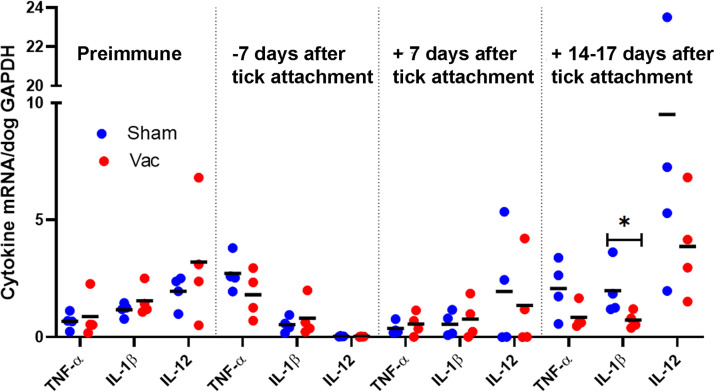
Vaccination of dogs with rEtpE-C does not induce proinflammatory cytokines upon attachment of *E. chaffeensis-*infected ticks. Expression of mRNAs encoding TNF-α, IL-1β, and IL-12 was normalized by dog GAPDH mRNA in blood samples from four rEtpE-C-vaccinated and four sham-vaccinated dogs before vaccination, on day 7 before tick attachment and on day 7 and days 14 to 17 after attachment of *E. chaffeensis-*infected ticks (RT-qPCR). *, *P < *0.05; based on the unpaired two-tailed Student’s *t* test. Horizontal bars indicate mean values.

IFN-γ is a good indicator of a protective, cell-mediated immune response to *E. chaffeensis* infection (15, 27, 28). The ELISpot assay allows the direct quantification of individual cytokine-secreting cells *ex vivo* based on capture ELISA, and the sensitivity of an ELISpot assay for cytokine detection in culture supernatants is 10- to 200-times greater than that of traditional ELISA (29). The canine IFN-γ ELISpot assay revealed that peripheral blood mononuclear cells (PBMCs) freshly collected from EtpE-C-vaccinated dogs significantly responded to rEtpE-C stimulation by secreting IFN-γ at 1 week before and 1 week after attachment of infected ticks, whereas PBMCs from sham-vaccinated dogs did not respond to rEtpE-C stimulation 1 week before tick attachment and only weakly responded 1 week after attachment (Fig. 6A). The difference between rEtpE-C-vaccinated and sham-vaccinated dogs was statistically significant (*P < *0.001), as assessed with a negative binomial mixed model, with similar differences between the groups for data obtained at 1 week before and 1 week after tick attachment. RT-qPCR revealed that IFN-γ mRNA in buffy coat peripheral blood leukocytes was significantly upregulated in vaccinated dogs at day 7 and during days 14 to 17 after attachment of infected ticks compared with the sham-vaccinated dogs (Fig. 6B). Thus, vaccination with rEtpE-C induced a significant memory T-cell response that could thwart a challenge with *E. chaffeensis-*infected ticks. rEtpE-C vaccination itself did not induce a significant proinflammatory cytokine response (mRNA for tumor necrosis factor alpha [TNF-α], interleukin 1β [IL-1β], or IL-12) in any of the dogs. After a challenge with infected ticks, there was a tendency for vaccination to reduce the weak proinflammatory cytokine, although the differences in mRNA levels were not significant except for IL-1β mRNA at 14 to 17 days post-tick challenge (Fig. 7).

### EtpE-C vaccination of dogs does not reduce *E. chaffeensis* infection or EtpE expression in transmission-fed ticks.

**FIG 8:**
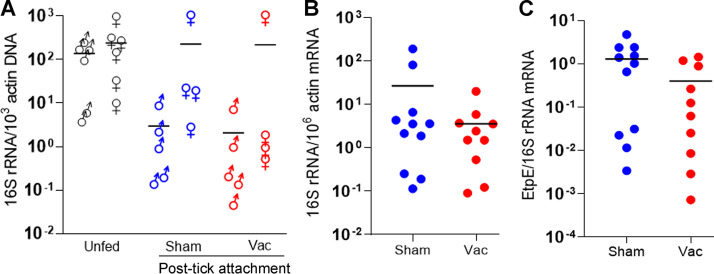
EtpE-C-vaccination of dogs does not reduce *E. chaffeensis* infection or EtpE expression in transmission-fed ticks. (A) Expression of the 16S rRNA gene of *E. chaffeensis* in male and female ticks (normalized by *A. americanum* tick actin DNA) before and after feeding on rEtpE-C-vaccinated or sham-vaccinated dogs (qPCR). (B) Expression of 16S rRNA by *E. chaffeensis* (normalized by *A. americanum actin* mRNA) in the salivary glands of female ticks removed from rEtpE-C-vaccinated and sham-vaccinated dogs (RT-qPCR). (C) Expression of *EtpE* mRNA by *E. chaffeensis* (normalized by *E. chaffeensis* 16S rRNA) in the salivary glands of female ticks removed from rEtpE-C-vaccinated and sham-vaccinated dogs (RT-qPCR).

qPCR revealed that the ratio of the *E. chaffeensis* 16S rRNA gene (reflecting *E. chaffeensis* abundance) to *A. americanum* actin DNA in each of the male and female whole ticks did not differ significantly between vaccinated and sham-vaccinated dogs (Fig. 8A). Thus, the feeding of ticks on dog blood containing anti-EtpE-C did not reduce *E. chaffeensis* replication in infected adult ticks. Similarly, RT-qPCR revealed that the levels of *E. chaffeensis* 16S rRNA and *A. americanum actin* mRNA in salivary glands isolated from the attached female ticks did not differ significantly between vaccinated and sham-vaccinated dogs (Fig. 8B). Moreover, the ratio of *E. chaffeensis EtpE* mRNA to 16S rRNA in salivary glands from female ticks did not differ significantly between EtpE-C-vaccinated and sham-vaccinated dogs (Fig. 8C).

## DISCUSSION

Our results demonstrate that EtpE is expressed by *E. chaffeensis* in tick cells and that an antiserum against rEtpE-C significantly inhibits *E. chaffeensis* transmission from infected tick cells to human monocytes *in vitro*. This overall approach can be used to screen potential vaccine candidates for blocking the transmission of *E. chaffeensis* from ticks to mammals. Vaccination of dogs with rEtpE-C induced strong anti-EtpE-C antibodies and led to a rapid clearance of *E. chaffeensis* caused by a challenge with infected ticks, although vaccination did not block the initial transmission of *E. chaffeensis* from infected ticks to dogs. These results are similar to those obtained for dogs vaccinated with a live *E. chaffeensis* Himar1 transposon mutant ECH_0660 vaccine (30). Nonetheless, our rEtpE-C vaccine offers several advantages over live attenuated vaccines, including (i) obviating the chance for reversion to the wild-type bacteria or causing illness in immunosuppressed individuals, (ii) absence of inflammatory *Ehrlichia* components that may be present in live attenuated vaccines, (iii) cost-effectiveness and ease of preparation and storage, and (iv) vaccine stability. Hence, the EtpE-C could be used in a candidate vaccine for vaccination of humans to counter tick-transmitted ehrlichiosis.

IFN-γ-pretreated human monocytes are resistant to *E. chaffeensis* infection (27). The *E. chaffeensis* Arkansas strain induces a potent IFN-γ response in severe combined immunedeficient (SCID) mice, and this response suppresses *E. chaffeensis* infection; however, SCID mice infected with the *E. chaffeensis* Wakulla strain, which cannot induce IFN-γ, succumb to overwhelming infection (15, 28). Although subunit vaccines are generally considered ineffective for inducing cell-mediated immunity, our rEtpE-C vaccine administered with an ISCOM adjuvant induced an early IFN-γ response in dogs, similar to results obtained upon vaccination of dogs with live *E. chaffeensis* mutant ECH_0660 (30). Interestingly, using rEtpE-C as antigen, neither Western blotting nor ELISA detected any antibody response to rEtpE-C in sham-vaccinated animals challenged with *E. chaffeensis-*infected ticks. This is in agreement with our previous observation that sera from HME patients (naturally infected via tick bite) recognize rEtpE-N (N terminus of EtpE) more strongly than rEtpE-C (13), which may allow effective infection.

Blood is the sole source of nutrients for ticks. The volume of blood ingested ranges from 200- to 600-times an adult tick’s unfed body weight (31). Ticks concentrate blood meals by excreting waste products and lymph back into the host animal through the salivary glands (32), and thus, the salivary gland is the main route for the dissemination of tick-borne pathogens to mammals (33). Our results revealed that the relative amounts of *E. chaffeensis* in female and male transmission-fed ticks were similar to those of pretransmission-fed ticks despite the huge expansion of tick body mass, indicating rapid multiplication of *E. chaffeensis* in ticks after a blood meal. This expansion of the *E. chaffeensis* population in ticks was not significantly affected by the vaccination of dogs with rEtpE-C; furthermore, vaccination did not significantly affect the dispersal and/or growth of *E. chaffeensis* in tick salivary glands. This differs from results with Borrelia burgdorferi, which is destroyed in I. scapularis nymph ticks that have fed on mice vaccinated with recombinant outer surface protein A (OspA) of B. burgdorferi (34). Of the many differences between *Borrelia* sp. and *Ehrlichia* sp., the fact that *E. chaffeensis* is an obligatory intracellular bacterium may have precluded its neutralization by anti-rEtpE-C (acquired from dog blood) in the tick midgut lumen. Consequently, the earliest opportunity to prevent tick-to-mammal transmission of *E. chaffeensis* via vaccination with rEtpE-C is likely when *E. chaffeensis* is liberated from tick cells—perhaps at the site of tick bite.

How and when *E. chaffeensis* disperses from the tick midgut lumen to the salivary gland is unknown. Unlike B. burgdorferi, which moves from the midgut to the salivary gland (35) via the hemocoel in I. scapularis (35–37), the bacterium Rickettsia monacensis moves via the tracheal air tube in I. scapularis (38). For B. burgdorferi, dissemination to and infection of the salivary glands of I. scapularis nymphal ticks occurs 36 to 48 h after attachment (36). *E. chaffeensis* was found in the salivary gland of flat, molted adult ticks that had been infected as nymphs (19). In the present study, *E. chaffeensis* could already be detected in dog blood at 1-day post-tick attachment, which is much faster than what occurs with *Borrelia* sp.; thus, it is likely that *E. chaffeensis* spreads to tick salivary glands much more rapidly than B. burgdorferi.

We used engorged nymphal ticks to syringe inoculate *E. chaffeensis*, as originally reported by Karim et al. (39), with some modifications, as follows: semipurified host cell-free *E. chaffeensis* bacteria, instead of whole *E. chaffeensis-*infected DH82 cells (39), were used for the inoculation. This approach did not prevent ticks from molting, and *E. chaffeensis* was transstadially transmitted from the nymph stage to the adult stage. The infected adult ticks were competent to transmit *E. chaffeensis* to dogs, in agreement with a previous study (30). Thus, this method of preparing infected adult ticks is useful and more cost-effective and convenient compared with attaching naive nymphs to *E. chaffeensis-*infected dogs at the acute stage of infection (19).

Dogs and deer are the only experimental animal models currently available for tick-mediated transmission of *E. chaffeensis* (19, 20). To date, only the Arkansas strain was used in deer (40) and Arkansas and St. Vincent strains have been used for *E. chaffeensis* infection studies in dogs (19, 30, 41). Similar to what we observed in the present study, these strains cause long-lasting subclinical-to-mild disease in deer and dogs (19, 20, 30, 41–44). Thus, there is a need for highly virulent *E. chaffeensis* strains for dogs or other animal models for the purpose of evaluating vaccine efficacy for preventing severe HME caused *E. chaffeensis* transmitted by ticks.

Although dogs are not currently considered major reservoirs of *E. chaffeensis* infection, naive ticks can acquire *E. chaffeensis* from subclinically infected dogs and subsequently transmit *E. chaffeensis* upon biting naive dogs (19); thus, subclinically infected dogs can serve as competent reservoirs of *E. chaffeensis*. Vaccination of dogs is more feasible than vaccination of wild deer and may reduce the risk of tick-mediated transmission of *E. chaffeensis* from dogs to humans. Therefore, an EtpE-C-based vaccine is applicable to both humans and dogs to prevent the spread of *E. chaffeensis* infection to humans.

## MATERIALS AND METHODS

### Ethics statements.

All animal experiments were performed in accordance with The Ohio State University Institutional Animal Care and Use Committee guidelines and approved e-protocol. The university program has full continued accreditation by the Association for Assessment and Accreditation of Laboratory Animal Care International (AAALAC-I) under number 000028, dated 9 June 2000, and has a Public Health Services assurance, renewal number A3261-01, dated 6 February 2019 through 28 February 2023. The program is licensed by the U.S. Department of Agriculture, number 1-R-014, and is in full compliance with Animal Welfare Regulations.

### Preparation of *E. chaffeensis* cultures and host cell-free *E. chaffeensis*.

The canine macrophage cell line DH82 was used for culturing *E. chaffeensis* Arkansas (45) in Dulbecco’s minimal essential medium (DMEM; Mediatech, Manassas, VA) supplemented with 5% fetal bovine serum and 2 mM l-glutamine at 37°C in 5% CO_2_/95% air in a humidified atmosphere, as previously described (27). Cells were monitored for 2 to 3 days for infection using Hema 3 stain (Thermo Fisher Scientific, Waltham, MA) with centrifuged specimens and were passaged or harvested when the percentage of infected cells reached >95%, as described previously (27). *E. chaffeensis-*infected DH82 cells (∼1 × 10^8^ cells, >90% infected) were harvested by centrifugation at 400 × *g* for 5 min at 4°C. The pellet was resuspended in DMEM and sonicated on ice for 8 s at an output setting of 2 with a W-380 Sonicator (Heat Systems, Newtown, CT). Unbroken cells were removed by centrifugation at 1,000 × *g* for 5 min at 4°C, and the supernatant was passed through 5.0- and 2.7-μm GD/X nylon filters (Whatman, Florham Park, NJ) to remove cell debris and then centrifuged at 10,000 × *g* for 10 min at 4°C (46).

### Purification of recombinant EtpE-C and of rabbit anti-EtpE-C serum.

rEtpE-C was produced and purified as described previously (13), with minor modifications. Briefly, competent Escherichia coli BL21(DE3) cells (New England BioLabs, Ipswich, MA) transformed with a pET33b plasmid encoding EtpE-C were induced with 0.5 mM isopropyl-β-d-thiogalactopyranoside (Gold Bio, St. Louis, MO) at 30°C for 5 h, after which they were centrifuged, harvested, and lysed. After several washes, inclusions containing rEtpE-C were dissolved in 6 M guanidine hydrochloride and loaded onto a Poly-Prep chromatography column (Bio-Rad, Hercules, CA) containing HisPur cobalt resin (Thermo Fisher Scientific). The column was washed with 10 mM imidazole in 8 M urea, and bound proteins were eluted with 250 mM imidazole in 8 M urea. The purified proteins were suspended in SDS-PAGE sample buffer (50 mM Tris-HCl [pH 6.8], 2% SDS, 10% glycerol, 0.1% bromophenol blue, and 5% 2-mercaptoethanol), boiled for 5 min, and then separated using 10% SDS-PAGE. The SDS-PAGE gel was stained with GelCode blue (Thermo Fisher Scientific), destained with water, and imaged with a LAS3000 image documentation system (Fujifilm Medical Systems USA, Inc., Stamford, CT). The concentration of rEtpE-C was estimated with serial dilutions of bovine serum albumin (BSA; Thermo Fisher Scientific) as a standard. Antisera against rEtpE-C were produced in rabbits by Covance (Denver, PA) using purified rEtpE-C, and the anti-rEtpE-C IgG was affinity purified using the recombinant proteins by Covance.

### Tick cell culture and infection with *E. chaffeensis*.

The tick cell line ISE6 from embryos of I. scapularis was maintained in L15C300 medium (L15 medium [Difco, Detroit MI] supplemented with 5% each of fetal bovine serum and tryptose phosphate broth [Difco] and 0.2% bovine lipoprotein cholesterol concentrate [MP Biomedicals, Irvine, CA; hereafter, tick basic medium]) at 34°C, as described previously (47). Infection with *E. chaffeensis* requires constant neutral pH, which was achieved by further supplementing with tick basic medium supplemented with 25 mM HEPES [pH 7.5], additional 5% fetal bovine serum, and 0.25% NaHCO_3_ (hereafter tick *Ehrlichia* medium) (47). The host cell-free *E. chaffeensis* pellet derived from one T75 flask was resuspended in 1-ml tick basic medium, and 500 μl was added to a 30% to 50% confluent tick cell culture in 2 wells in a 24-well plate. Infection of ISE6 cells with *E. chaffeensis* was monitored every 2 days using cytocentrifuged slides stained with Hema 3.

### Detection of native EtpE in ISE6 cells.

Western blotting and immunofluorescence microscopy were performed as described previously (13). *E. chaffeensis*-infected tick cell lysates and rEtpE-C were subjected to SDS-PAGE (10% polyacrylamide gels), and the separated protein bands were transferred to a nitrocellulose membrane and then subjected to Western blotting with rabbit polyclonal antibodies against rEtpE-C, *E. chaffeensis* outer membrane protein P28 (48), and tick actin (as a tick cell loading control; Sigma, St. Louis, MO). Immunopositive bands were detected using horseradish peroxidase-conjugated anti-rabbit IgG (KPL, now SeraCare, Gaithersburg, MD) and visualized with enhanced chemiluminescence by incubating the membranes with Pierce ECL Western blotting substrate (Thermo Fisher Scientific). Immunofluorescence microscopy was carried out with *E. chaffeensis*-infected DH82 or tick (ISE6) cells (80% to 90% infected cells). Cells were cytocentrifuged, fixed with 4% paraformaldehyde, and permeabilized with PGS (phosphate-buffered saline [PBS] with gelatin and saponin, composed of 137 mM NaCl, 2.7 mM KCl, 8.1 mM Na_2_HPO_4_, and 1 mM KH_2_PO_4_ [pH 7.4] supplemented with 0.1% gelatin and 0.3% saponin [both from Sigma]). Samples were incubated with rabbit anti-rEtpE-C serum (1:100), followed with Alexa Fluor 488-conjugated goat anti-rabbit IgG (Thermo Fisher Scientific), and imaged with a DeltaVision deconvolution microscope (Applied Precision, Issaquah, WA).

### Assessing the block of *E. chaffeensis* transmission *in vitro*.

Once infection with *E. chaffeensis* reached >60%; approximately 5 × 10^5^ *E. chaffeensis-*infected ISE6 cells in 3 wells of a 48-well plate were preincubated with rabbit preimmune or anti-EtpE-C serum (each diluted 1:32) in tick *Ehrlichia* medium at 34°C for 2 h, and 1 × 10^5^ to 2 × 10^5^ uninfected THP-1 cells were added and cocultured for additional 48 h in tick *Ehrlichia* medium at 34°C. The cells were cytocentrifuged, fixed with paraformaldehyde, permeabilized with PGS, labeled with mouse monoclonal anti-human CD147 (EMMPRIN; Santa Cruz Biotechnology, Santa Cruz, CA), followed by labeling with Alexa Fluor 488-conjugated goat anti-mouse IgG (Thermo Fisher Scientific) to distinguish human cells from ticks cells, and visualized with a DeltaVision microscope.

### Infection of cells with *E. chaffeensis* and analysis of ticks.

Freshly engorged nymphal *A. americanum* ticks were shipped to the lab overnight from the Oklahoma State University Tick Rearing Facility (Stillwater, OK). Upon arrival, ticks were cleaned with 70% ethanol and injected with host cell-free *E. chaffeensis* freshly isolated from DH82 cells (7 × 10^8^ to 10 × 10^8^ *Ehrlichia* per 2 to 4 μl, as assessed by qPCR) using a Hamilton syringe/needle (2.5 μl, Model 62 RN syringe and 33 gauge small-hub RN needle; Reno, NV). All injected ticks were maintained in an incubator with a 12 h dark/12 h light cycle at 25°C and >70% relative humidity (49) until they molted or were ready for dog challenge. Adult male and female ticks were tested for *E. chaffeensis* infection by qPCR (with DNA) or RT-qPCR (with RNA) prior to dog challenge. Ticks that had fed on dogs were pulled off the dogs after 10 to 13 days postinfestation. Salivary glands were isolated from partially or fully fed female ticks using a fine scalpel blade under a dissection microscope and saved in RNALater (Qiagen, Germantown, MD) at –80°C for RNA extraction. Freshly molted individual adult ticks were used to extract DNA using a DNA blood and tissue extraction kit (Qiagen).

### ISCOM preparation.

ISCOM with rEtpE-C was prepared as described previously (50) with slight modifications. Briefly, purified EtpE-C (1 ml; 0.5 to 1 mg/ml) that had been eluted from the affinity-purification column with 8 M urea was dissolved in 1 ml of 20% Mega 10 (*N*-decanoyl-*N*-methylglucamine; Sigma) and mixed with 100 μl of 1 mg/ml of cholesterol (Sigma), 1 mg/ml phospholipid (l-α-phosphatidylcholine), and 50 μl of 100 mg/ml Quil A (InvivoGen, San Diego, CA). The mix was rotated for 2 h at room temperature and sonicated in a water bath three times for 15 min each. The mix was placed in dialysis tubing (10 kDa MW cutoff; Spectrum, New Brunswick, NJ) and dialyzed against PBS for 24 h at room temperature and dialyzed again for 24 h at 4°C. ISCOM with PBS was prepared as a negative control, and ISCOM preparations were stored at –80°C or used directly for vaccinations.

### Vaccination of dogs and challenge with infected ticks.

Ten specific-pathogen-free Beagle dogs, age 1 to 2 years, were purchased from Covance and housed in the University Lab Animal Resources, College of Veterinary Medicine, The Ohio State University. Five dogs each were vaccinated with rEtpE-C ISCOM or PBS-ISCOM by subcutaneous injection to the subscapularis region on both sides (0.5 ml each side) three times with a 2-week interval. Two sites at the subscapularis area were shaved and bathed, and a 2-inch tubular stockinette cotton roll (Medichoice, Mechanicsville, VA) was glued with Animal ID tag cement (Nasco, Fort Atkinson, WI) to each site (two stockinettes per dog). *E. chaffeensis-*infected ticks (20 females, 10 males) were placed in each stockinette and allowed to feed until they started dropping off (12 to 15 days). Blood samples (5 to 8 ml) were collected from the saphenous vein. An aliquot of the whole blood (400 μl) was saved for DNA isolation, and buffy coat from 5 ml of blood was used for RNA isolation; plasma was saved for ELISA or Western blotting to determine the anti-EtpE-C titer. A 1-ml blood sample was used for a complete cell count or blood chemistry analysis at the Department of Clinical Pathology, The Ohio State University teaching hospital. The complete cell count and blood chemistry profiles were obtained for each dog before vaccination, after the third vaccination, and at 1 and 3 weeks after attachment of infected ticks.

### Isolation of dog PBMCs and ELISpot assay.

PBMCs were isolated using Ficoll-Paque Plus (GE Healthcare, Piscataway, NJ). Briefly, 5 ml of EDTA-treated whole blood was diluted 1:2 with RPMI 1640 medium (Gibco, Gaithersburg, MD) at room temperature, layered slowly over 5 ml Ficoll-Paque Plus in a 15-ml tube, and centrifuged at 400 × *g* for 30 min at 20°C. The PBMC layer was collected and washed twice with RPMI 1640 (along with centrifugation at 350 × *g* for 5 min), and PBMCs were resuspended in 1 ml RPMI 1640 containing 10% fetal bovine serum and a mixture of 1% l-glutamine and 1% antibiotic (Gibco). The ELISpot assay was performed using the canine IFN-γ ELISpot kit (R&D Systems, Minneapolis, MN). Briefly, 5 × 10^5^ PBMCs/well were plated in a canine IFN-γ-coated 96-well plate. The cells were stimulated with rEtpE-C (1 μg) or culture medium as a negative control. The immunopositivity of spots was assessed with an ImmunoSpot analyzer (Cellular Technology, Shaker Heights, OH).

### Titration of the anti-EtpE-C using ELISA.

The wells of a 96-well flat-bottom microtiter plate (Nunc MaxiSorp) were coated with 2 μg each of rEtpE-C and BSA in coating buffer (14 mM Na_2_CO_3_ and 34 mM NaHCO_3_ [pH 9.6]). The coated wells were blocked with 1% BSA in PBS for 1 h at 37°C. Serial 2-fold dilutions of preimmune or anti-EtpE-C serum from rabbits or 1:3,200 dilutions of sera from sham- or rEtpE-C-vaccinated dogs were incubated for 1 h at 37°C, followed by incubation with horseradish peroxidase-conjugated anti-rabbit IgG (KPL) or anti-dog IgG (KPL) for 1 h at 37°C. A TMB core+ (Bio-Rad) substrate solution was used to develop reactions for 10 to 15 min, and reactions were stopped by addition of 50 μl of 0.2 M sulfuric acid. Absorbance was measured at 450 nm in a SpectraMax Plus 384 microplate reader (Molecular Devices, San Jose, CA).

### Extraction of total RNA from *E. chaffeensis-*infected ISE6 cells, ticks, and dog blood.

ISE6 cells (1 × 10^6^ to 2 × 10^6^) at 3 days postinfection with host cell-free *E. chaffeensis* (isolated from DH82 cell cultures) were harvested and stored in 300 μl of RNAprotect reagent (Qiagen) at –20°C until RNA isolation. Individual ticks (male or female) after molting or being removed from dogs, tick salivary glands, or buffy coats collected from dog blood were used to isolate total RNA using the RNeasy minikit (Qiagen). cDNA was synthesized from 1 μg of extracted RNA using the Maxima H minus first-strand cDNA synthesis kit (Thermo Fisher Scientific) with random hexamer primers.

### RT-qPCR and qPCR.

RT-qPCR and qPCR were performed with cDNA or DNA, respectively, using gene-specific primers (see Table S1 in the supplemental material). Each PCR mix (25 μl) contained 250 nM of each primer, 12.5 μl of Maxima SYBR green/ROX qPCR Master Mix (2×) (Thermo Fisher Scientific), and 2.5 μl of cDNA template. Each PCR mix was subjected to the following thermal cycling conditions in an Mx3000P instrument (Stratagene, La Jolla, CA): 95°C for 10 min, followed by 40 cycles of 95°C for 30 s, 55°C for 1 min, and 72°C for 1 min. To quantify *Ehrlichia* sp., an absolute quantification method was used by creating a standard curve of the *Ehrlichia* 16S rRNA cloned into plasmid pUC19 as a standard template for qPCR (51). Relative expression ratios of a target gene compared with a reference gene (16S rRNA/tick actin, EtpE/16S rRNA, and dog cytokines/GAPDH) were estimated by the standard method (52).

10.1128/mBio.00895-20.1 TABLE S1 Primers used for qPCR and RT-qPCR. Download Table S1, DOCX file, 0.02 MB. Copyright © 2020 Budachetri et al. 2020 Budachetri et al. This content is distributed under the terms of the Creative Commons Attribution 4.0 International license.

### Statistical analysis.

Statistical analyses were performed with the unpaired two-tailed Student’s *t* test or analysis of variance (ANOVA) for continuous outcomes, as applicable. For the *E. chaffeensis* 16S rRNA *C_T_* data, *C_T_* values of >45 were capped and treated as censored values. A censored-data Cox model with random effects to account for repeated measures on dogs was used to assess differences between rEtpE-C-vaccinated and sham-vaccinated dogs. Differences in spot-forming units between rEtpE-C-vaccinated and sham-vaccinated dogs were assessed using a negative binomial generalized linear mixed model. A *P* value of <0.05 was considered significant. All graphs and statistical calculations were prepared with Prism 8 software (GraphPad, San Diego, CA) or R version 3.6.1, with the coxme package (26) for the censored-data Cox model and the lme4 package (53) for the negative binomial mixed model.
