# Supplementary material for: An Entry-Triggering Protein of Ehrlichia Is a New Vaccine Candidate against Tick-Borne Human Monocytic Ehrlichiosis
Source: mBio. 2020 Jul 28;11(4):e00895-20. doi: 10.1128/mBio.00895-20 (PMC7387794; doi:10.1128/mBio.00895-20)
Supplement: TABLE S1 [file mBio.00895-20-st001.docx]

## Table S1. Primers used for qPCR and RT-qPCR

| **Primer name** | **Target gene**  **(accession number)** | **Primer sequence (5'-3')** | **Amplicon size** | **References** |
| --- | --- | --- | --- | --- |
| KM0125 | *E. chaffeensis* 16S rRNA (NR_074500.2) | CGGGGGAAAGATTTATCGCTATTA | 323 | (1) |
| KM0126 |  | CGCTTGCCCCCTCCGTATTA |  |  |
| KB015 | *E. chaffeensis* EtpE  (NC_007799.1) | AGGGATTACAAGCAGTCAAGAG | 131 | This study |
| KB016 |  | TACGATGCCCACCAACTAATG |  |  |
| KB007 | *A. americanum* actin  (EZ000248.1) | CCGTGAGAAGATGACCCAAA | 123 | This study |
| KB008 |  | CCGGAGTCGAGCACAATAC |  |  |
| KB009 | *I. scapularis* actin  (XM_002413517.1) | CTTGGAGGGAGACATCTTTGTG | 111 | This study |
| KB010 |  | CGCTCCATGTCATTCCAATCT |  |  |
| KB011 | Human actin  (NM_001101.4) | AGAGCTACGAGCTGCCTGAC | 184 | (2) |
| KB012 |  | AGCACTGTGTTGGCGTACAG |  |  |
| KB025 | Dog GAPDH  (NM_001003142) | ATCACTGCCACCCAGAAGAC | 133 | (3) |
| KB026 |  | TCAGCTCAGGGATGACCTTG |  |  |
| KB027 | Dog IFN-γ  (NM_001003174) | GCGCAAGGCGATAAATGAAC | 82 | (3) |
| KB028 |  | CTGACTCCTTTTCCGCTTCC |  |  |
| KB029 | Dog IL-1β  (NM_001037971) | CAAGTCTCCCACCAGCTCTGTA | 81 | (3) |
| KB030 |  | GGGCTTCTTCAGCTTCTCCAA |  |  |
| KB031 | Dog IL-12  (NM_001003292) | CAGCAGAGAGGGTCAGAGTGG | 109 | (3) |
| KB032 |  | ACGA CCTCGATGGGTAGGC |  |  |
| KB033 | Dog TNF-α  (NM_001003244) | TCTCGAACCCCAAGTGACAAG | 153 | (3) |
| KB034 |  | CAACCCATCTGACGGCACTA |  |  |

References:

1. Wang X, Rikihisa Y, Lai TH, Kumagai Y, Zhi N, Reed SM. 2004. Rapid sequential changeover of expressed *p44* genes during the acute phase of *Anaplasma phagocytophilum* infection in horses. Infect Immun 72:6852-9.

2. Song Y, Zuo Y. 2014. Occurrence of HHIP gene CpG island methylation in gastric cancer. Oncol Lett 8:2340-2344.

3. Tamura Y, Ohta H, Yokoyama N, Lim SY, Osuga T, Morishita K, Nakamura K, Yamasaki M, Takiguchi M. 2014. Evaluation of selected cytokine gene expression in colonic mucosa from dogs with idiopathic lymphocytic-plasmacytic colitis. J Vet Med Sci 76:1407-10.
